# Eating and Drinking

**Published:** 1882-08

**Authors:** 


HOW TO MAKE STARCH FOR SHIRT-BOSOMS. — "Take two ounces of fine gum arabic powder, put it into a pitcher, and pour on a pint or more of boiling water, according to the strength you desire, and then, having covered it, let it stand all night; in the morning pour it carefully from the dregs into a clean bottle, cork it and keep it for use. A table-spoonful of gum-water stirred into a pint of starch made in the usual manner, will give to lawn, either white or printed, a look of newness, when nothing else can restore them after they have been washed."

I believe that unwarranted and monstrous errors are propagated by different writers, on the subject of food and drink. Each man has a whim or hobby, so that it has at length come to the point that if a man will live healthfully to a great age, say a hundred and fifty or two hundred years, he must eat nothing but grapes and drink nothing but rain-water. The gentleman who advocates the grape diet contends that wheat bread ought not to be eaten—that it has too much earth in it, and tends to stiffen a man's joints and muscles half a century sooner than if he subsisted on grapes.

